# SLC20A2-Associated Idiopathic basal ganglia calcification (Fahr disease): a case family report

**DOI:** 10.1186/s12883-022-02973-y

**Published:** 2022-11-17

**Authors:** Meiying Li, Qin Fu, Liangxu Xiang, Yingwei Zheng, Wenjing Ping, Yongjun Cao

**Affiliations:** 1 Department of Neurology, Ma’anshan Hospital, 243099, Ma’anshan, China; 2 Department of Neurology, The Second Affiliated Hospital of Soochow University, 215004 Suzhou, China

**Keywords:** Fahr disease, Idiopathic basal ganglia calcification (IBGC), SLC20A2

## Abstract

**Background:**

Idiopathic basal ganglia calcification (IBGC) is a genetic disorder of the nervous system commonly known as Fahr disease. IBGC patients with a genetic background are considered to have primary familial brain calcification (PFBC), also known as familial basal ganglia calcification (FBGC), or familial Fahr disease. It is a rare degenerative neurological disorder characterized by extensive bilateral basal ganglia calcification that can lead to a range of extrapyramidal symptoms and neuropsychiatric manifestations. Studies have suggested that more than 50 variants of SLC20A2 gene mutations account for approximately 50% of IBGC cases. There is a wide spectrum of mutation types, including frameshift, nonsense, and splice site mutations in addition to deletion and missense mutations. Here we report a case of familial basal ganglia calcification caused by a frameshift mutation in the SLC20A2 gene. We identified a heterozygous mutation in the SLC20A2 gene, c.1097delG (p.G366fs*89). To our knowledge, this mutation site has not been reported before.

**Case presentation:**

A 57-year-old male patient was admitted to the hospital with “unstable walking and involuntary movements between the eyes and eyebrows for 6 months”. Based on the patient’s family history, symmetrical calcification foci in the bilateral caudate nucleus head, thalamus, cerebellum and parietal lobe indicated by head CT, and gene test results, the diagnosis of familial Fahr disease caused by mutations in the SLC20A2 gene, c.1097delG p.G366fs*89) was confirmed.

**Conclusion:**

For the first time, we identified c.1097delG (p.G366fs*89) as a frameshift mutation in the IBGC family. This frameshift mutation caused the condition in this family of patients. This mutation not only broadens the range of known SLC20A2 mutations but also aids in the genetic diagnosis of IBGC.

## Background

Idiopathic basal ganglia calcification (IBGC) is a genetic disorder of the nervous system characterized by calcification of the basal ganglia and other parts of the brain, commonly known as Fahr disease. IBGC patients with a genetic background are considered to have primary familial brain calcification (PFBC),also known as familial basal ganglia calcification (FBGC), or familial Fahr disease [1]. Fahr disease clinically manifests as a variety of motor and cognitive disorders, such as Parkinson’s disease, dystonia, migraine, dementia, psychosis and emotional symptoms [2]. It is a kind of pedigree disease with heterogeneous clinical and pathogenic genes. Since 2012, studies have reported that variations in several different genes contribute to the disease, with autosomal dominant inheritance patterns including SLC20A2 [3], PDGFRB [4], PDGFB [5] and XPR1 [6]. There are also partial autosomal recessive patterns of genes such as MYORG [7] and ,more recently, JAM2 [8]. SLC20A2 gene mutation is believed to be responsible for most IBGC families thus far [9]. This gene encodes type III sodium phosphate transporter 2 (PiT2), whose loss-of-function mutation may lead to local accumulation of inorganic phosphate in the brain, leading to calcium phosphate deposition [10].

SLC20A2 gene mutations have a wide range of mutation types, including deletion mutations, missense mutations, frameshift mutations, nonsense mutations and splice site mutations. This article describes a case of familial Fahr disease most likely caused by a novel frameshift mutation in SLC20A2 (c.1097delG p.G366fs*89).

## Case presentation

**Fig. 1:**
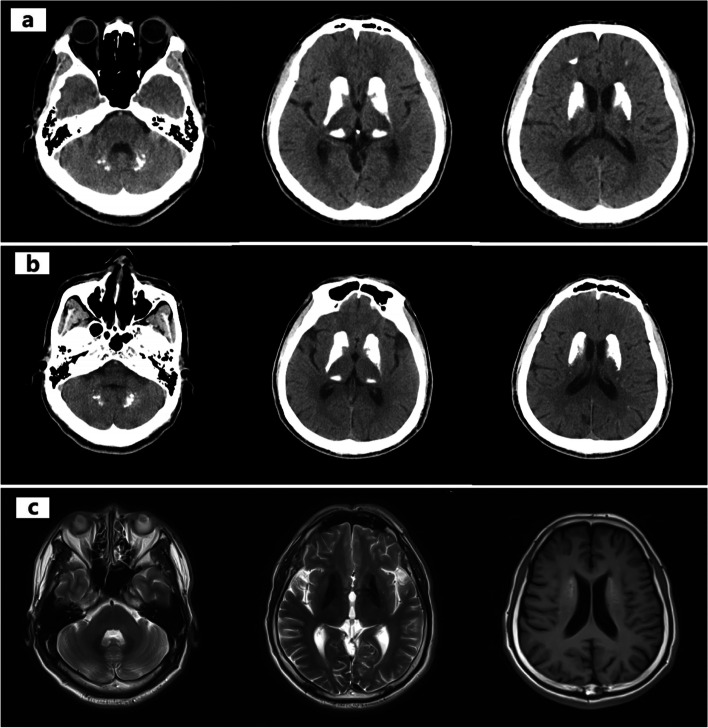
CT and MRI of the proband (II: 3). **a** and **b** were taken on 2020/08/04 and 2021/05/28, respectively. Both CT scans showed multiple calcifications in the head of the bilateral caudate nucleus, bilateral thalamus, bilateral cerebellum, and bilateral parietal lobes, and there was no significant difference between the two lesions. **c** shows the MRI. Symmetrical abnormal signals in the bilateral dorsal thalamus, basal ganglia and dentate nucleus of the cerebellar hemisphere are considered to be paramagnetic deposits

The proband (II: 3, male, 57 years old) had a history of diabetes mellitus and found multiple intracranial calcifications for 3 years. More than 6 months before admission, the patient began to consciously walk unsteadily, and the symptoms gradually worsened, with obvious shaking when standing and mild weakness of limbs. Two weeks before, the patient showed decreased language fluency and conscious unresponsiveness. Nervous system examination revealed involuntary eyelid movement, weakened limb muscle strength, normal muscle tone, absence of limb tendon reflexes, positive sign of difficulty in standing with eyes closed, inability to walk in a straight line, and wide-based gait. The rest of the physical examination was negative. Complete clinical blood tests showed no obvious abnormalities. Head CT showed multiple calcifications in the head of the bilateral caudate nucleus, bilateral thalamus, bilateral cerebellum, and bilateral parietal lobes (Fig. 1a and b). Symmetrical abnormal signals in the bilateral dorsal thalamus, basal ganglia, and dentate nucleus of the cerebellar hemisphere were observed on plain MR scans of the head, suggesting that paramagnetic deposition was considered (Fig. 1c).

**Fig. 2:**
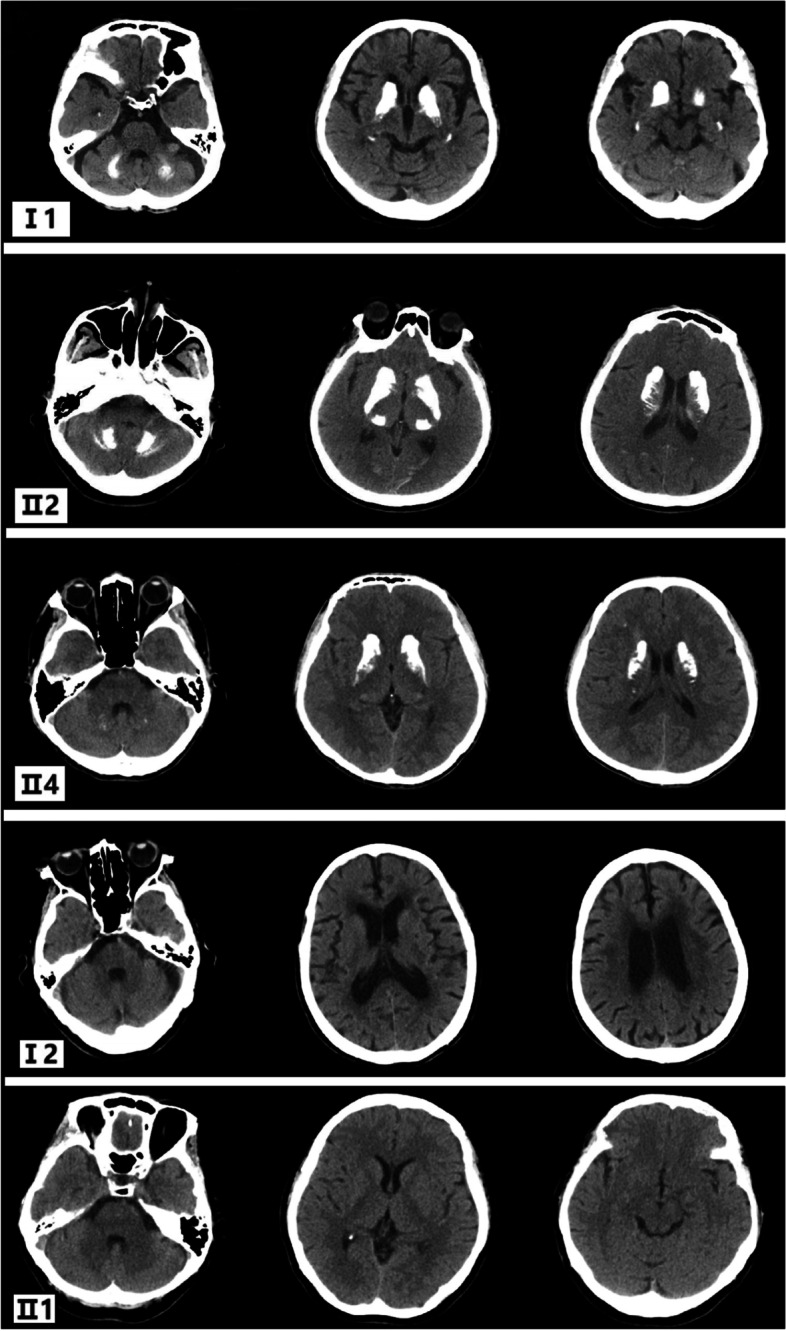
CT report of the proband’s family. The CT scans of his father (I: 1), second sister (II: 2) and younger brother (II:4) showed multiple intracranial calcifications. The CT scan of her mother (I: 2) and elder sister (II: 1) showed no obvious abnormality

**Fig. 3:**
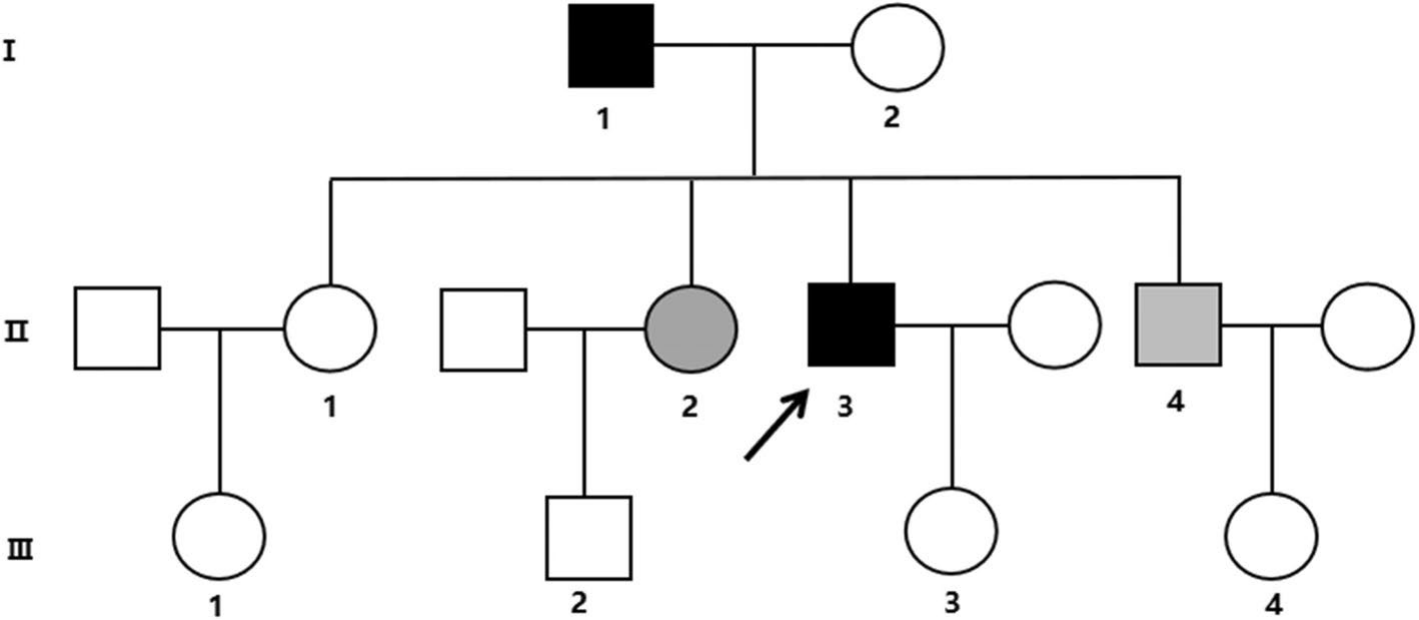
Molecular genetic test report. The proband (II: 3), father (I: 1), second sister (II: 2) and younger brother II:4) share heterozygous mutations in the SLC20A2 gene (c.1097delG p.G366fs*89) and the mother (I: 2) and the elder sister(II: 1) was wild-type

**Fig. 4:**
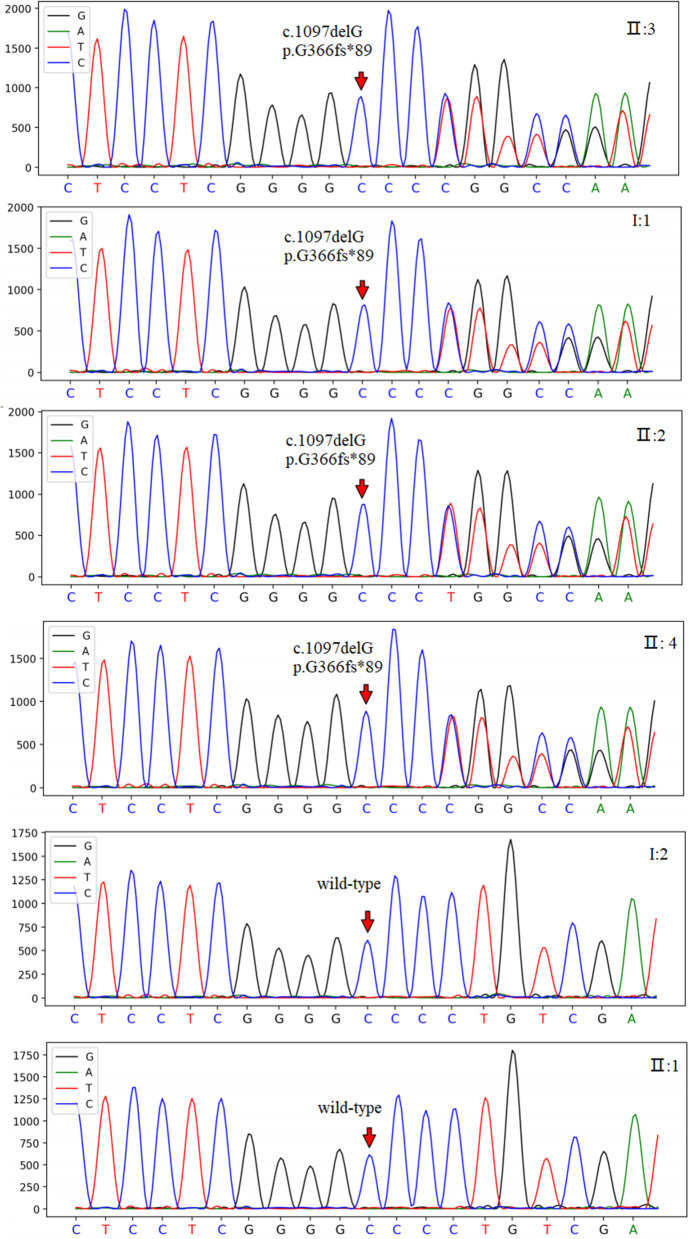
Pedigree of the proband family: the box represents male. The circle represents a female. The filling black represents symptomatic patients. The filled grey represents asymptomatic patients and the arrow points to the proband

Supplementary examination of serum calcium, inorganic phosphorus, parathyroid hormone, free metanephrine, and free methoxynorepinephrine were normal. Tracing the patient’s medical history, we learned that the proband’s father (I:1) also had similar extrapyramidal symptoms while the proband’s mother (I:2), eldest sister (II:1), second sister (II:2), and younger brother (II:4) did not have any clinical symptoms. We further improved the head CT of this family, and the results showed that multiple intracranial calcifications were seen in the proband’s father (Fig. 2I:1), second sister (Fig. 2 II:2) and younger brother (Fig. 2 II: 4), while the head CT of his mother (Fig. 2I:2) and elder sister (Fig. 2II:1) showed no obvious abnormalities. Combined with the patient’s clinical symptoms, family history, head CT findings of the bilateral caudate nucleus head, thalamus, cerebellum, parietal lobe and other symmetrical calcification foci, and no obvious abnormality in the clinical blood test, our preliminary diagnosis was considered familial basal ganglia calcification. To confirm the diagnosis, we mobilized the patient to improve the genetic testing of the family. The test revealed that the proband had a disease-causing mutation in the SLC20A2 gene. The chromosome position of this mutation is CHR8:42294933 (genome version: HG19), transcript NM_001257180, located in the 8th exon, which is a frameshift mutation (Fig. 3 II:3). This heterozygous mutation came from the father of the proband (Fig. 3I:1), and this mutation was also detected in the second sister (Fig. 3 II:2) and younger brother (Fig. 3 II:4), who had calcifications on head CT, while the gene detection of the mother (Fig. 3I:2), and the elder sister (Fig. 3 II:1) was wild-type. The SLC20A2 gene-related reported disease is an autosomal dominant inheritance, and this heterozygous mutation is consistent with the disease-dominant inheritance pattern (Fig. 4). The gene-related disease was consistent with the clinical manifestations of the patient, and the second sister and brother also had calcifications on head CT. Although there are no clinical symptoms, this heterozygous mutation may lead to the occurrence of the disease.

## Genetic analysis

At the first step of genetic investigations, patient blood samples were collected in EDTA-containing tubes. Genomic DNA was extracted from whole blood. Individual whole-exome sequencing of the patient’s DNA was performed to investigate aetiological genomic variants. KAPA Library Preparation Kit reagent was used to construct a DNA library. The program includes three steps: standard DNA repair and at the end of a - tail, adapters, and amplification. Purification was performed with Agencourt AMPure XP beads between steps. The captured DNA samples were then subjected to Illumina NovaSeq high-throughput sequencing. Sequencing data were obtained via Illumina Sequence Control Software (SCS) after assessment was eligible, data read and bioinformatics analysis.

Combined with the results of genetic testing, the proband found a pathogenic mutation in the SLC20A2 gene. The chromosomal location of the variant is CHR8:42294933 (genomic version: HG19), and the transcript NM_001257180, located in exon 8, is a frameshift mutation (c.1097delG p. G366fs *89). It is not included in the HGMD professional database and Clinvar database. The population frequency of this locus was not recorded. It is not included in the gnom AD database for East Asian populations. This variant was also detected in the second sister and younger brother, who both had calcification on head CT. According to the ACMG gene mutation interpretation guidelines, this locus was in accordance with 3 pieces of evidence (PVS1, PM2, PP1). This variant was classified as a pathogenic variant.

Given that the variant was a frameshift mutation, no software was available to predict evolutionary conservation and protein structure or function. We regret that due to the limited platform, we are unable to further verify site-related functional verification by cell or animal experiments. We also expect to be able to verify the mutation site-related function through some basic studies, such as cell or animal experiments in the future.

## Discussion

Here, we first report a case of familial basal ganglia calcification potentially caused by a frameshift mutation in SLC20A2 (c.1097delG p.G366fs *89). IBGC is a genetic disorder of the nervous system, commonly known as Fahr disease, characterized by calcification of the basal ganglia and other parts of the brain. It is a rare degenerative neurological disease characterized by extensive bilateral basal ganglia calcification, which on CT shows symmetrical calcification in the caudate nucleus, globus pallidus and dentate nucleus and may be accompanied by calcification in the white matter, thalamus, cortex, midbrain, pons and other parts of the brain, while other parts are rarely involved [1]. Clinical manifestations vary according to the different sites involved, including extra pyramidal symptoms (increased muscle tone, tremor, dance signs, oral-facial movement disorders, etc.), cognitive disorders, mental symptoms, gait, language, pyramidal tract signs, ataxia, epilepsy, sensory disorders, etc. [2].

Fahr disease is a kind of pedigree disease with heterogeneous clinical and pathogenic genes. Most of the cases are familial, with an autosomal dominant (AD) pattern of inheritance. The first pathogenic gene, SLC20A2, was discovered by Wang et al. in 2012. The SLC20A2 gene is located at 8p21.1-8q11.23, contains 11 exons, and encodes type III sodium-phosphorus cotransporter 2 (PiT2). PiT2 is a multitransmembrane protein composed of 652 amino acids with dual functions of sodium and phosphorus cotransport and retroviral receptors [3]. SLC20A2 mutation can cause PiT2 phosphorus transport disorder, resulting in increased local Pi concentration and inducing vascular smooth muscle cells or other types of cells to differentiate into osteoblast-like cells through the effect of PiT1, leading to vascular calcification. On the other hand, SLC20A2 mutation may combine with Ca in the matrix to form calcium phosphate deposits in the interstitial space. Pathological studies have shown that calcium deposition in the basal ganglia is the primary cause of radiological manifestations of the disease. Mucoglycan, trace amounts of aluminium, arsenic, cobalt, copper, molybdenum, iron, lead, manganese, magnesium, phosphorus, silver, and zinc are also deposited there [8]. After the discovery of the first causative gene SLC20A2, a large number of subsequent studies have found other genes with autosomal dominant inheritance patterns, including PDGFRB, PDGFB and XPR1, and genes in the autosomal recessive inheritance pattern MYORG and JAM2. Among them, autosomal dominant cases can cause disease through abnormal inorganic phosphorus transport, disruption of the blood-brain barrier and pericyte homeostasis. The pathogenesis of autosomal recessive cases is unknown, but functional tests have confirmed the pathogenicity. For example, the MYORG-encoded product is involved in myoblast differentiation and regulates the glycosylation of endoplasmic reticulum proteins in astrocytes; the JAM2-encoded product is highly expressed in neurovascular unit-related cells and is involved in intercellular adhesion, regulation of cell polarity and endothelial cell permeability, and maintenance of blood-brain barrier function [4–8].

The clinical diagnostic criteria of Fahr disease were first proposed by Moskowitz et al., and subsequently revised by Manyam et al. [11], 1989 and 2005. The current diagnostic criteria are as follows:

(1) Neuroimaging demonstrated bilateral calcification of the basal ganglia. Other brain regions may also be observed. (2) Progressive neurological dysfunction, usually involving motor impairment and/or neuropsychiatric manifestations. (3) Age of onset is typically in the fourth or fifth decade, although dysfunction can also appear in childhood. (4) No biochemical abnormalities or physical features suggested mitochondrial or metabolic disease or other systemic diseases. (5) Absence of an infectious, toxic, or traumatic cause. (6) Family history consistent with autosomal dominant inheritance.

Fahr disease is an exclusive diagnosis. This diagnosis can be considered when imaging suggests symmetrical brain calcifications in the basal ganglia and exclusion of secondary factors. Secondary factors to be excluded include but are not limited to the following: ① abnormal calcium and phosphorus metabolism: such as hypoparathyroidism and pseudohypoparathyroidism; ② brucella, HIV, Toxoplasma gondii and other infections. ③ lead, carbon monoxide and other poisoning; ④ systemic lupus erythematosus and other autoimmune disease;. ⑤cockayne syndrome, Aicardi-Goutières syndrome, Coat’s syndrome and other genetic diseases [12].

In this case, the proband fully met the diagnostic criteria. Combined with the genetic test results, the proband had a disease-causing mutation in the SLC20A2 gene. The chromosome position of this mutation is CHR8:42294933 (genome version: HG19), transcript NM_001257180, located in the 8th exon, which is a frameshift mutation. SLC20A2 gene reported disease is idiopathic basal ganglia calcification type 1 (213,600, AD), autosomal dominant inheritance. The clinical symptoms can be urinary incontinence, mask-like face, psychosis, depression, dysarthria, intellectual decline, gait instability, Parkinson’s disease, dystonia, tremor, hyperreflexia, tonic, bradykinesia, dance-like movement, alternate movement disorder, basal ganglia calcification, postural instability, headache, memory impairment, poor limb distance discrimination, dense calcification of cerebellum dentate nucleus, small blood vessel calcification, adult onset, progressive/progressive, pyramidal dysfunction. The phenotype of the proband was consistent with the phenotype of the disease, and the genealogy of the proband was consistent with the genetic rule of dominant disease. IBGC is a slowly progressive disease that can last for many years. The clinical manifestations of patients can be divided into two stages: the first is the formation of intracranial calcification, and the second is the occurrence and development of corresponding clinical symptoms caused by intracranial calcification, but not all patients will show clinical symptoms. In this case report, the second sister (II:2) and younger brother (II:4) of the proband are carriers of the mutation. There is calcification on head CT, and there are currently no clinical symptoms. With increasing age, the disease may progress, clinical symptoms may appear, and follow-up should be strengthened.

Regrettably, considering that screening asymptomatic individuals has no direct medical benefit to adults or young adults, and may lead to psychological harm, we speculate that the next generation of young people has a certain probability of inheritance. Thus, far, there are no clinical symptoms, so we did not screen the next generation of young people in this family, which is the shortcoming of this paper [13].

At present, there is no specific treatment for Fahr disease, mainly symptomatic treatment, including antipsychotic drugs, antidepressants, antiepileptics and precognitive drugs [14]. When patients develop psychiatric syndromes, other diseases must be considered. Identifying secondary causes with potentially correctable abnormalities may allow some symptoms to be resolved (e.g., correcting phosphate and calcium levels in hypoparathyroidism), thereby preventing disease progression and improving prognosis [15]. It is expected that there will be more in-depth research on the pathogenesis and pathogenesis of Fahr disease in the futur to provide a safer and more effective treatment plan for clinical treatment of Fahr disease.

## Conclusion

For the first time, we identified c.1097delG (p.G366fs*89) as a frameshift mutation in the IBGC family. This frameshift mutation most likely caused the condition in this family of patients. This mutation not only broadens the range of known SLC20A2 mutations, but also aids in the genetic diagnosis of IBGC.

## Data Availability

Data sharing is not applicable to this article as no datasets were generated or analysed during the current study.

## Abbreviations

IBGC: Idiopathic basal ganglia calcification
FBGC: familial idiopathic ganglia calcification
PFBC: Primary familial brain calcification
CT: computed tomography
AD: Autosomal dominant
AR: Autosomal recessive
MRI: magnetic resonance imaging
PiT: phosphate transporter
SCS: Sequence Control Software
